# Supplementary material for: G Protein-Coupled Receptor Kinase 2 Selectively Enhances β-Arrestin Recruitment to the D2 Dopamine Receptor through Mechanisms That Are Independent of Receptor Phosphorylation
Source: Biomolecules. 2023 Oct 20;13(10):1552. doi: 10.3390/biom13101552 (PMC10605370; doi:10.3390/biom13101552)
Supplement: Supplementary file 1 [file biomolecules-13-01552-s001.zip › biomolecules-2659732-supplementary.pdf]

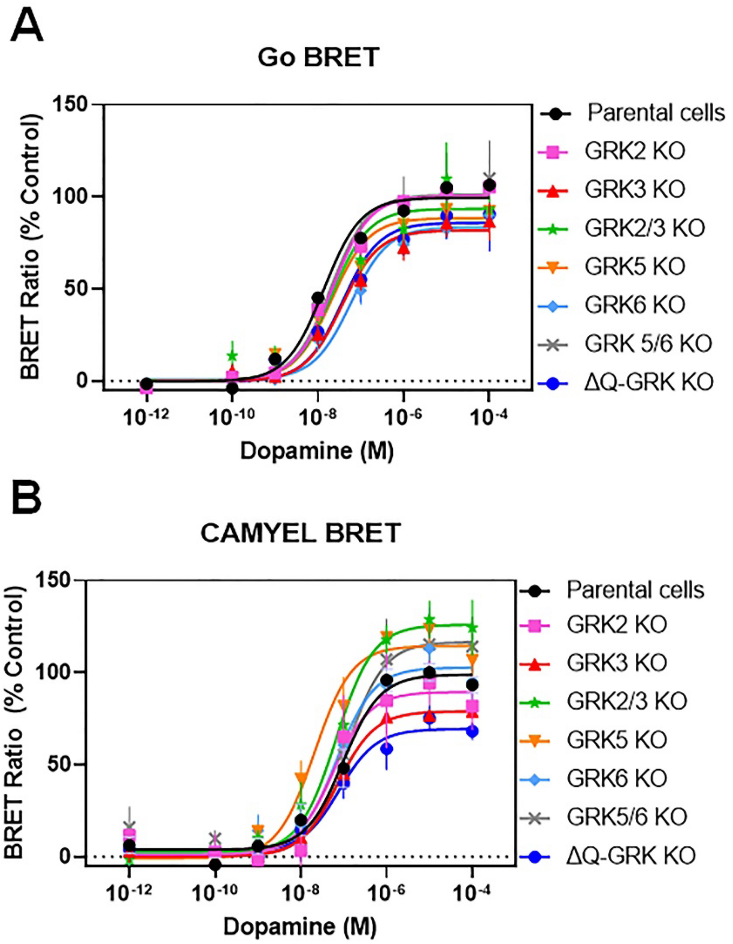

Figure S1. GRK knockout does not affect D2R-mediated G protein signaling. D2R-mediated Go BRET and CAMYEL BRET assays were performed as described in Section 2 using parental HEK293 cells or HEK293 cells in which the indicated GRK isoforms were deleted (KO) using CRISPR/Cas9 technology (Note ΔQ-GRK KO refers to the simultaneous deletion of GRK2, GRK3, GRK5, and GRK6). **(A)** Concentration-response curves for dopamine stimulation of Go activation were generated as described in Section 2. **(B)** Concentration-response curves for D2R-mediated inhibition of forskolin-stimulated cAMP accumulation using the CAMYEL BRET assay were performed as described in Section 2. In both panels, the data are expressed as a percentage of the maximum dopamine response observed in the parental HEK293 cells and are displayed as mean ± SEM values from at least three experiments. Average curve parameters (EC_{50} and Emax values) and statistical comparisons are shown in **Table S1**.

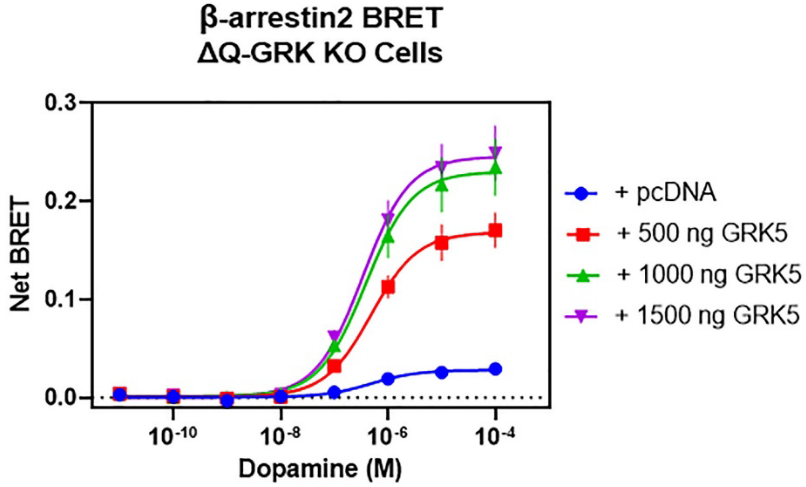

Figure S2. Overexpression of GRK5 produces maximal β-arrestin2 recruitment to the D2R in GRK KO cells with no effect on dopamine potency. The indicated quantities (ng DNA) of plasmid expressing GRK5 were transiently transfected into the ΔQ-GRK KO cell line and dopamine-stimulated β-arrestin2 recruitment was measured as described in **Figure 2C** and Section 2. Net BRET refers to the change in BRET signal following dopamine stimulation. Data are expressed as the mean ± SEM net BRET of at least three independent experiments. The curve parameters are as follows: + pcDNA (control): EC_{50} = 500 ± 88 nM, Emax = 0.0278 ± 0.0035; + 500 ng GRK5: EC_{50} = 480 ± 34 nM, Emax = 0.168 ± 0.0189**; + 1000 ng GRK5: EC_{50} = 380 ± 34 nM, Emax = 0.229 ± 0.0297***; + 1500 ng GRK5: EC_{50} = 340 ± 28 nM, Emax = 0.245 ± 0.0262***. Statistical comparisons between pcDNA control and experimental condition were performed using a one-way ANOVA with Dunnett's multiple comparisons test: *$p < 0.05$; **$p < 0.005$; ***$p < 0.001$.

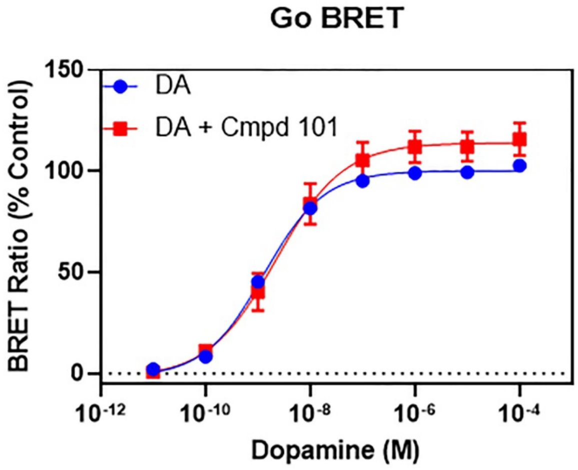

Figure S3. Compound 101 has no effect on D2R-mediated Go protein activation. D2R-mediated Go BRET assays were performed as described in Section 2 using parental HEK293 cells. Concentration-response curves for dopamine stimulation of Go activation were generated as described in Section 2. The data are expressed as a percentage of the maximum dopamine response and are displayed as mean ± SEM values from at least three experiments. Average (mean ± SEM) curve parameters (EC_{50} and Emax values) and statistical comparisons are as follows (Emax = % parental control): DA (control): EC_{50} = 1.4 ± 0.02 nM, Emax = 100; DA + Cmpd 101: EC_{50} = 3.0 ± 1.5 nM, Emax = 114 ± 7.8. Statistical differences between the curve parameters in the presence or absence of compound 101 were assessed using a t-test and found not to be significant ($p > 0.05$).

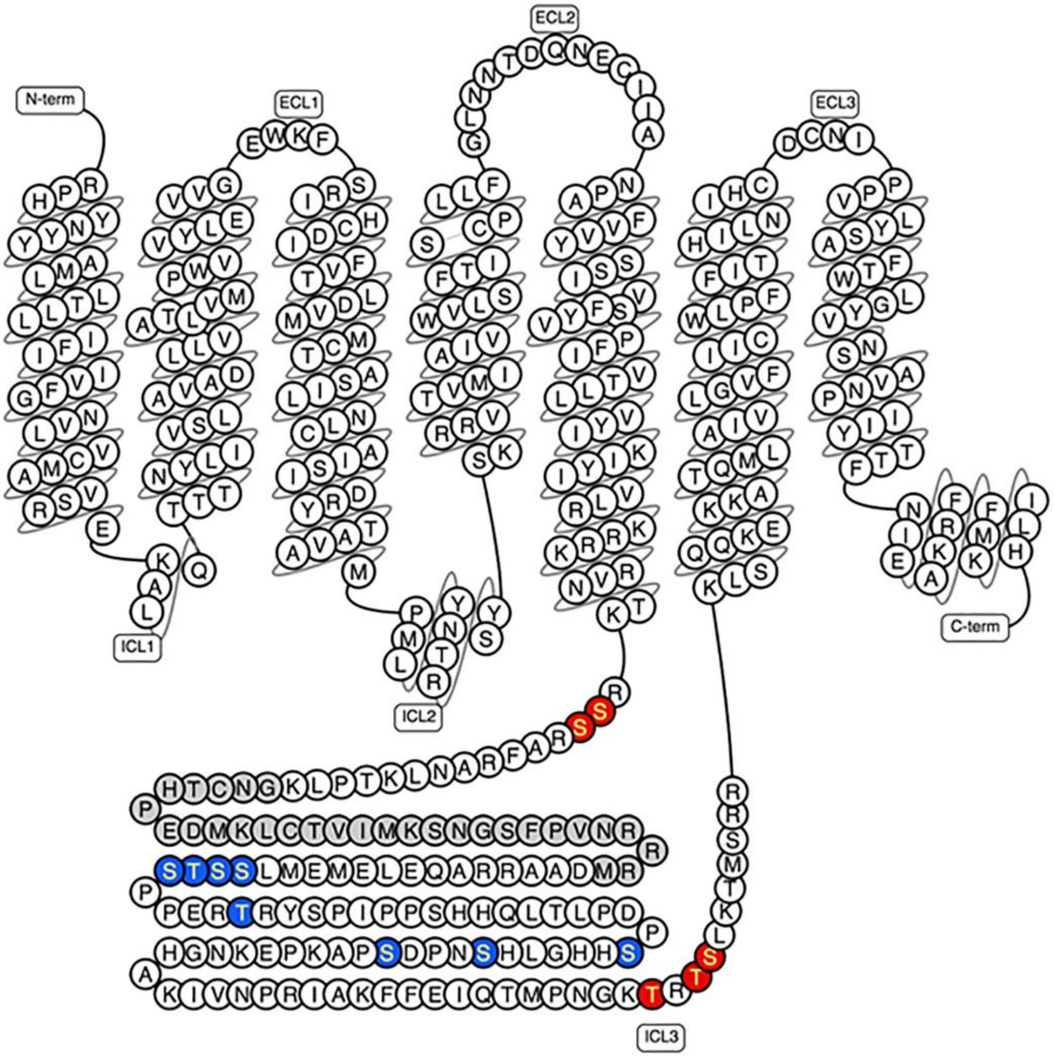

Figure S4. Diagram of the rat D2R illustrating the phosphorylation-deficient mutant receptor used in this study. PKC and GRK phosphorylation sites are highlighted in red and blue, respectively, and were identified as described in Namkung et al. 2004 [38], and Namkung et al., 2009 [17]. Grey residues highlight the 29-amino acid region present in the D2R long isoform. Mutation of all the PKC and GRK phosphorylation sites from wild-type serines and threonines to alanines and valines, respectively, in order to create a completely phosphorylation-deficient D2R (PO_4-null D2R) was described in Namkung et al., 2009 [17]. The D2R snake diagram was generated using GPCRdb [53].

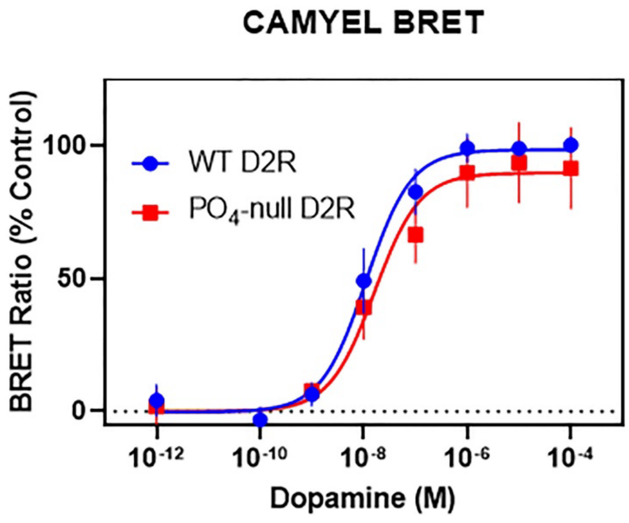

Figure S5. The PO_4-null D2R signals through G proteins in a normal fashion. WT and PO_4-null D2R were transfected into parental HEK293 cells and concentration-response curves for dopamine-mediated inhibition of forskolin-stimulated cAMP accumulation using the CAMYEL BRET assay were generated as described in Section 2. Data are expressed as a percentage of the maximum response elicited by WT D2R and are shown as mean ± SEM of at least three experiments performed in triplicate. The curve parameters are as follows (Emax = % parental control): WT D2R EC_{50} = 19 ± 12 nM, Emax = 100; PO_4-null D2R: EC_{50} = 28 ± 16 nM, Emax = 91 ± 13. The WT and PO_4-null D2R curve parameters were compared statistically using a t test and were found not to differ ($p > 0.05$).

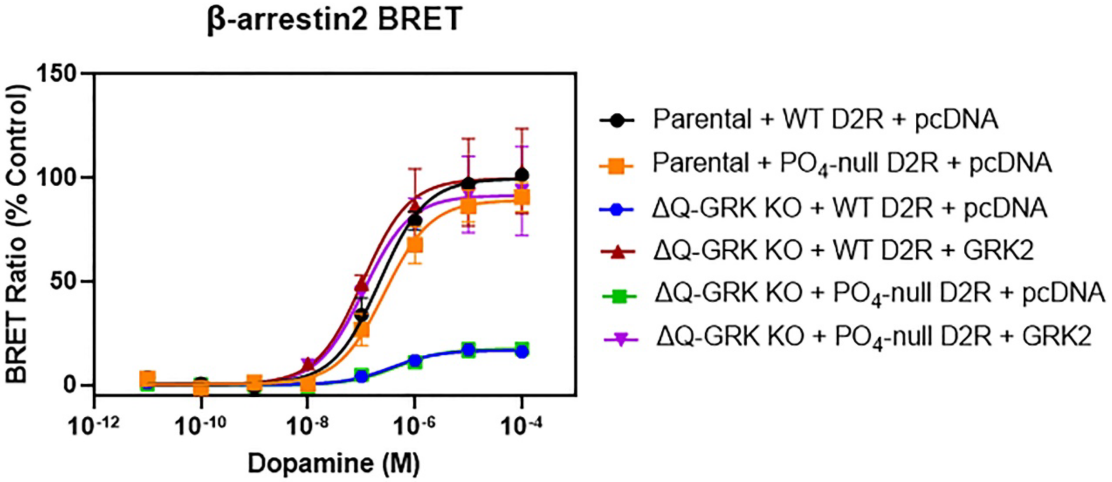

Figure S6. The effect of GRK2 rescue in the ΔQ-GRK KO cells does not differ between the WT and PO_4-null D2R. The WT and PO_4-null D2R were transfected with or without GRK2 into either parental HEK293 cells or HEK293 cells lacking all GRK expression (ΔQ-GRK KO cells). Concentration-response curves for dopamine-stimulated β-arrestin2 recruitment were generated as described in Section 2. The curve parameters are as follows (Emax = % parental control): Parental + WT D2R + pcDNA: EC_{50} = 250 ± 99, Emax = 100; Parental + PO_4-null + pcDNA: EC_{50} = 330 ± 130 nM, Emax = 90 ± 6.8; ΔQ-GRK KO + WT D2R in + pcDNA: EC_{50} = 350 ± 70 nM, Emax = 17 ± 1.6; ΔQ-GRK KO + WT D2R + GRK2: EC_{50} = 100 ± 27 nM, Emax = 100 ± 22; ΔQ-GRK KO + PO_4-null D2R + pcDNA: EC_{50} = 450 ± 140 nM, Emax = 17 ± 3.3; ΔQ-GRK KO + PO_4-null D2R + GRK2: EC_{50} = 110 ± 45 nM, Emax = 92 ± 20. The WT and PO_4-null D2R curve parameters in each condition (Parental + pcDNA, ΔQ-GRK KO + pcDNA, and ΔQ-GRK KO + GRK2) were compared statistically using a one-way ANOVA with Sidak's's multiple comparisons test and found not to be significant ($p > 0.05$).

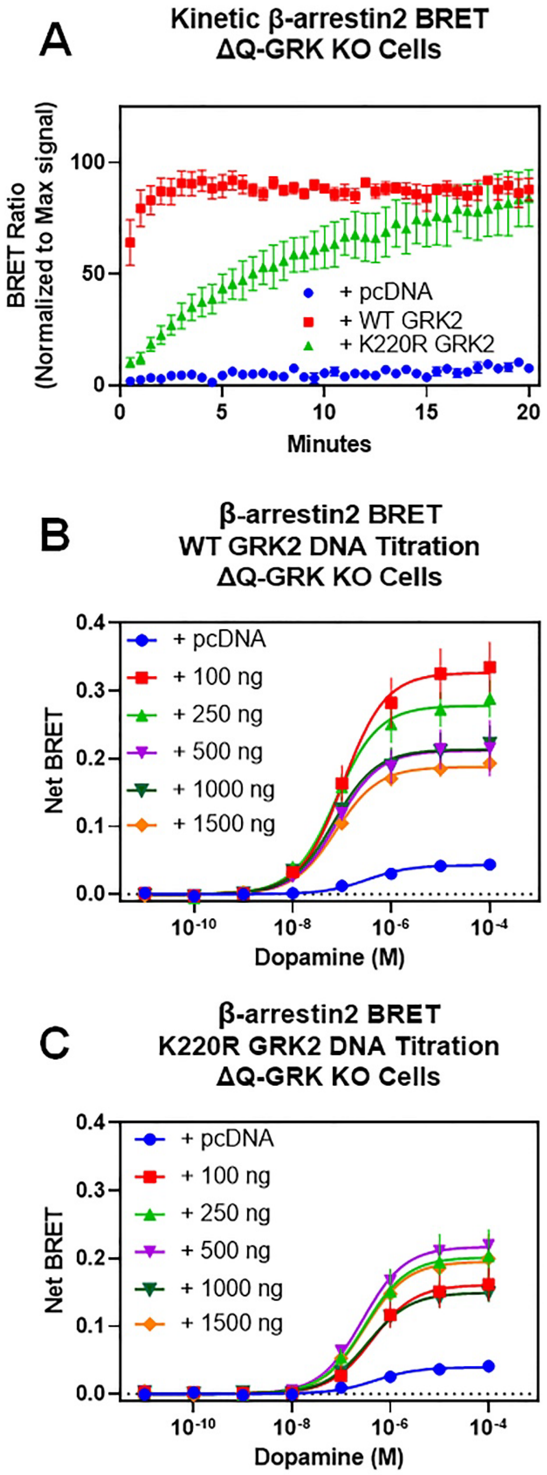

Figure S7. The effects of the K220R GRK2 mutant on dopamine-stimulated β-arrestin2 recruitment to the D2R. β-arrestin2 BRET assays were performed as described in Section 2 using the WT D2R-Rluc8 transfected into the ΔQ-GRK KO cells. **(A)** Cells were transfected with 1000 ng

each of WT GRK2, K220R GRK2, or pcDNA (control) and then stimulated with 100 μM dopamine at time zero. D2R-β-arrestin2 BRET was then measured as a function of time up to 20 min. The data represent the mean ± SEM values from three experiments and are normalized to the maximum BRET signal observed in each assay. **(B)** The indicated quantities (ng DNA) of plasmid expressing WT GRK2 were transiently transfected into the ΔQ-GRK KO cell line and dopamine-stimulated β-arrestin2 recruitment to the WT D2R was measured as described in Section 2. Net BRET refers to the change in BRET signal following dopamine stimulation. Data are expressed as the mean ± SEM net BRET of three independent experiments. The curve parameters are as follows: + pcDNA (control) EC_{50} = 340 ± 80 nM, Emax = 0.043 ± 0.004; + 100 ng EC_{50} = 110 ± 13 nM**, Emax = 0.327 ± 0.037****; + 250 ng EC_{50} = 78 ± 11 nM***, Emax = 0.278 ± 0.026****; + 500 ng EC_{50} = 87 ± 21 nM***, Emax = 0.199 ± 0.021**; + 1000 ng EC_{50} = 71 ± 6.4 nM***, Emax = 0.213 ± 0.026**; + 1500 ng EC_{50} = 77 ± 3.2 nM***, Emax = 0.188 ± 0.012**. **(C)** The indicated quantities (ng DNA) of plasmid expressing the K220R GRK2 were transiently transfected into the ΔQ-GRK KO cell line and dopamine-stimulated β-arrestin2 recruitment to the WT D2R was quantitated. Data are expressed as the mean ± SEM net BRET of at least three independent experiments. The curve parameters are as follows: + pcDNA (control) EC_{50} = 590 ± 150 nM, Emax = 0.0396 ± 0.008; + 100 ng EC_{50} = 450 ± 84 nM, Emax = 0.161 ± 0.023*; + 250 ng EC_{50} = 320 ± 44 nM, Emax = 0.202 ± 0.040**; + 500 ng EC_{50} = 270 ± 26 nM, Emax = 0.217 ± 0.005**; + 1000 ng EC_{50} = 360 ± 77 nM, Emax = 0.149 ± 0.014*; + 1500 ng EC_{50} = 320 ± 42 nM, Emax = 0.195 ± 0.035**. Statistical comparisons between pcDNA control and the experimental conditions were performed using a one-way ANOVA with Dunnett's multiple comparisons test: *$p < 0.05$; **$p < 0.005$; ***$p < 0.001$; ****$p < 0.0001$.

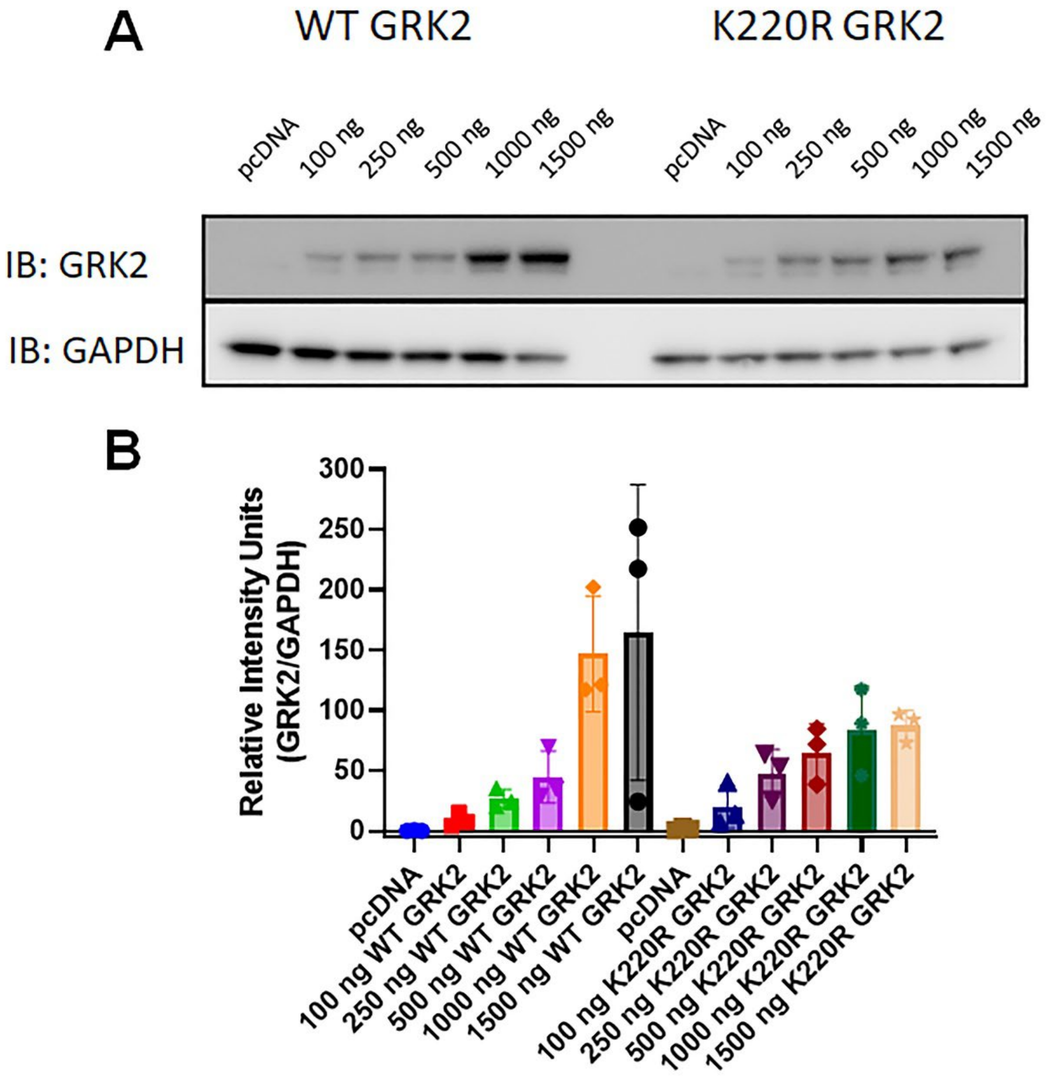

Figure S8. Evaluation of the cellular expression of the WT GRK2 and the K220R mutant GRK2 via Western blotting. (**A**) ΔQ-GRK KO cells were transfected with WT D2R-Rluc8, β-arrestin2-mVenus, and the indicated amount (ng) of GRK2 construct or pcDNA control. Proteins were extracted, separated by SDS-PAGE, and immunoblotted (IB) for either GRK2 or GAPDH (loading control) as described in Section 2. Blots from a representative experiment (that was performed 3 times) are shown. (**B**) The GRK2 expression obtained in **A** was quantified using ImageJ software. Data are presented as the relative intensity units (GRK2/GAPDH) and expressed as mean ± SEM. The WT GRK2 and K220R GRK2 expression using equivalent quantities of transfected DNA were compared using a one-way ANOVA with Sidak's multiple comparisons test and found not to be significant in all cases ($p > 0.05$).

Table S1. GRK knockout does not affect D2R-mediated G protein signaling. Curve parameters are derived from **Figure S1**. EC_{50} and Emax values represent the mean ± SEM of the maximal dopamine response in the HEK293 parental cells.

**Dopamine-Stimulated**		**Parental HEK293**	**GRK2 KO**	**GRK3 KO**	**GRK2/3 KO**	**GRK5 KO**	**GRK6 KO**	**GRK 5/6 KO**	**ΔQ GRK KO**
G_o BRET	EC_{50} (nM)	16 ± 2.7	29 ± 7.0	33 ± 6.3	20 ± 5.3	96 ± 81	67 ± 27	23 ± 6.8	41 ± 6.2
	E_{max} (% Parental)	100	101 ± 3.1	82 ± 7.4	93 ± 11	91 ± 5.7	84 ± 5.3	102 ± 20	86 ± 13
CAMYEL BRET	EC_{50} (nM)	180 ± 75	49 ± 16	92 ± 31	100 ± 49	46 ± 18	110 ± 63	91 ± 41	260 ± 200
	E_{max} (% Parental)	100	94 ± 12	80 ± 2.1	130 ± 12	120 ± 6.0	104 ± 17	121 ± 16	71 ± 3.8

Statistical comparisons between the HEK293 parental curve parameters (pEC_{50} values were used for statistical analyses) and the GRK KO experimental groups were made using a one-way ANOVA with Dunnett's multiple comparisons test and found not to be significant in all cases ($p > 0.05$).
